# Family and College Environmental Exposures Mediate the Relationship between Parental Education and Depression among College Students

**DOI:** 10.1371/journal.pone.0151759

**Published:** 2016-03-18

**Authors:** Hui Zhai, Lu Chen, Yanjie Yang, Hailian Sun, Hui Pan, Jincai He, Xiongzhao Zhu, Hong Sui, Wenbo Wang, Xiaohui Qiu, Zhengxue Qiao, Xiuxian Yang, Jiarun Yang, Yunmiao Yu, Bo Ban, Changzhi He

**Affiliations:** 1 Department of Medical Psychology, Public Health Institute of Harbin Medical University, Harbin, China; 2 Department of Endocrinology, Peking Union Medical College Hospital, Beijing, China; 3 Department of Clinical Psychology, First Affiliated Hospital of Harbin Medical University, Harbin, China; 4 Department of Internal Neurology, First Affiliated Hospital of Wenzhou Medical University, Wenzhou Medical College, Wenzhou, China; 5 Department of Medical Psychology, Second Xiangya Hospital, Central South University, Changsha, China; 6 Department of Epidemiology and Health Statistics, Public Health Institute of Harbin Medical University, Harbin, China; 7 Department of Endocrinology, Affiliated Hospital of Jining Medical College, Shandong, China; Penn State College of Medicine, UNITED STATES

## Abstract

**Background:**

Depression is a major health concern for college students due to its substantial morbidity and mortality. Although low parental education has been identified as a factor in depression in college students, the mechanisms through which parental educational achievement affects students’ depression are not well understood. We tested whether adverse family and college environments mediate the relationship between parental educational level and depression among Chinese college students.

**Methods:**

A total of 5180 respondents were selected using a cross-sectional survey. We examined the association of parental education, adverse family and college environments with depression in college students using the Adolescent Self-Rating Life Events Checklist, Beck Depression Inventory and socio-demographic questionnaires.

**Results:**

Lower parental educational level is significantly correlated with depression in college students in our sample. Additionally, low family economic status, paternal or maternal unemployment, long periods spent apart from family, family conflicts, having been scolded and beaten by parents, poor or dissatisfying test performance, conflict with friends, heavy course load and failure in selection processes are also associated with parental education. Low family economic status, paternal or maternal unemployment, long periods spent apart from family, family conflicts, poor or dissatisfying test performance, conflict with friends and heavy course load mediated the relationship between parental education and depression in college students.

**Conclusions:**

Adverse family and college environments could explain the influence of parental educational level on depression in college students.

## Introduction

Lower socioeconomic status is associated with poorer health, including depression [1]. Depression is common among higher-education students [2]. The prevalence of depression in college students is high, at approximately 15% [3]. A large number of depressive college students experience adverse consequences, including suicide, which is the leading cause of death among college students [4]. According to the American College Health Association, 10% of college students have experienced suicidal ideation, mostly as a result of depression. Among college students who commit suicide, 95% have significant depression [5]. For this reason, effective interventions to prevent depression in college students should be prioritized as a public-health issue. Depression studies should not neglect socioeconomic status as a factor, due to its importance as a predictor of depression.

Depressive symptoms are more commonly observed among families with a lower socioeconomic status, as has been shown by many previous surveys [6]. Families with a lower socioeconomic status are often exposed to economic hardships, experience a large family burden and find themselves in a serious family conflict situation as the children grow up [7]. Therese concluded that children under disadvantageous family conditions may experience increased mental distress. Parental education can effectively affect children’s perceived level of social support and normal development [8]. Low social support has been shown to exert a direct impact on family strain and leads to a poor coping response and depression. Decreased parental education influences developmental abnormalities among children who often present with nutritional deficiencies and poorer moods [9].

Previous studies have demonstrated that the influence of environment on health varied across levels of socioeconomic status [10]. Individuals whose parents have low educational attainment experience a threefold risk of depression [11]. Among college students, the living environment is mostly composed of their family and their college. Although family environment may have a strong mediating effect, the college environment should not be neglected. We discuss the potential roles of adverse family and college environments in the relationship between parents’ educational level and depression in college students. Adverse family factors associated with depression in college students included low family economic status, parental divorce, poor parental relationships, paternal or maternal unemployment, long periods spent apart from family, family conflicts and having been scolded and beaten by parents. Adverse factors in the college environment correlated with depression in college students included to dissatisfaction with their major, poor or dissatisfying test performance, conflict with friends, heavy course load, tension with teachers, failure in selection processes, transfer to another school or having quit school and experiencing pressure to re-enroll. Most of these factors may be connected to lower parental educational levels.

Our study will suppose a mediated model among parental educational level, adverse family and college environment and depression in college students. There exists (a) the relationship between parental educational level and adverse family and college environment; (b) the link between parental educational level and depression in college students; (c) the mediation effect from parental educational level to adverse family and college environment to depression in college students.

## Materials and Methods

### Subjects and procedure

Participants were selected in the city of Harbin, in the North of China, where there are 14 universities, three of which are national key universities and eleven of which are ordinary universities. To obtain a representative sample of Chinese university students, we selected two national key universities (Harbin Institute of Technology and Harbin Engineering University) and four ordinary universities (Harbin Medical University, Harbin Normal University, Heilongjiang University and Heilongjiang Institute of Technology). The latter were chosen in a random draw. We calculated that our analysis would require 1800 national key university students and 4200 ordinary university students, estimating sample size depending on the number of students in each university. Stratified sampling was used to select full-time students. The samples were then stratified into first-year, second-year, third-year, fourth-year and postgraduate-year students. Investigators went into classes to distribute questionnaires. Every participant consented to the survey before beginning. The research described in this paper meets the ethical guidelines of the ethics committee of Harbin Medical University. Approval for this study was granted by the Ethics Committee of Harbin Medical University. All the participants were explained the nature and purposes of the study. Participation was completely voluntary, with no economic or other motivation, and each participant signed a written statement of informed consent.

### Measures

We collected information on gender, age, university type, whether the student was an only-child, smoking and alcohol-consumption habits and insomnia using a self-composed socio-demographic questionnaire. Information on maternal and maternal educational levels (primary school or below, junior middle school, senior high school and university or above) was also requested. Family economic status (good, medium or bad), parental status (married or divorced), parental relationship (good, medium or bad) and dissatisfaction with their chosen major (satisfaction, quite satisfied or basically satisfied) were calculated.

Other family and school environments were tested by the Adolescent Self-Rating Life Events Checklist (ASLEC) [12]. The questionnaire consisted of 27 items intended to measure the intensity of life events and stress (none, mild, moderate, severe, extremely severe). We used items 8, 17 and 24 to assess whether students spent long periods apart from their families and the presence of family conflicts and scolding/beating by parents. Items 3, 4, 9, 10, 18, 19, 20 and 22 tested whether students experienced poor or dissatisfying test performance, conflict with friends, heavy course load, tension with teachers, failure in selection processes, transfer to another school or having quit school and pressure to enter school.

Depression levels were assessed by the Beck Depression Inventory (BDI), which included 21 items with scores ranging from 0–3[13]. Each item addressed a mental-health state or related symptoms, including mood, pessimism, feeling of failure, dissatisfaction, sense of having sinned, sense of being punished, self-loathing, self-blame, suicidal ideation, weepiness, irritability, social withdrawal, procrastination, body-image distortion, inactivity, dyssomnia, fatigue, appetite loss, weight loss, body preoccupation and loss of sexual desire. The total score represents the degree of depression. The higher the score, the more severe the depression. Scores lower than 5 were considered to indicate no depression, whereas scores of 5–13 indicated mild depression, 14–20 indicated moderate depression and more than 20 indicated severe depression. BDI, which is one of the most commonly used self-rated depression scales, can be adapted to every age group and is considered highly reliable. Specifically, it can be adapted to all adult age ranges as well as to children and adolescents. Since the year 1976, BDI has been generally used for surveys with more than 600 samples, no in matter normal groups or diagnosed patient groups[14].

### Data analytic plan

We examined factors related to the family and college environment as mediators between parental educational level and depression. SPSS 18.0 and Bootstrap for SPSS were used to analysis the data. The detailed process was as follows:

First, the distribution of parental educational level with regard to every factor related to family and college environment was assessed using descriptive statistics. Second, we used the χ^2^ test and t test to identify differences in gender, age, university type, only-child status, smoking, alcohol-consumption, symptoms of ill health, insomnia and adverse family and college environmental factors across all levels of parental education. Then, we examined the effects of parental educational level using a logistic regression analysis, with depression as the dependent variable when controlling for gender, age, university type, only-child status, smoking and alcohol-consumption habits, symptoms of ill health and insomnia. We carried out a second regression analysis to examine the effects of parental educational level on all significant exposure environments into regression. A Sobel test was used to examine the roles of adverse family and college environments in the logistic regression model.

## Results

We handed out 6000 questionnaires to the college students in Harbin. The response rate was 91.3%. After questionnaires with missing responses were eliminated, 5180 remained. There were 2643 (51.0%) male and 2537 (49%) female respondents. Slightly more males than females responded, in accordance with the gender distribution of the schools. The average age of the participants was 21.32 years (SD = 2.20), ranging from 16 to 43. The sample distributions across university types, only-child status, smoking and alcohol-consumption habits, symptoms of ill health and insomnia and various adverse family and college factors are shown in Table 1. A total of 398 students (7.7%) reported a parental educational level of primary school or less. Additionally, the numbers of students reporting parental education levels of junior middle school, senior high school and university level or above were, respectively, 1202 (23.2%), 2156 (41.6%) and 1424 (27.5%). Additionally, the distributions of gender, age, university type, only-child status, smoking and alcohol consumption, symptoms of ill health, insomnia and the various adverse family and college factors for every parental educational level are also shown in Table 1. By contrast, from our evaluation of the socio-demographic variables and adverse family and college factors across parental educational levels, we concluded that parental educational level is related to gender, age, university type, only-child status, alcohol consumption, insomnia, low family economic status, paternal or maternal unemployment, long periods spent apart from family, family conflicts, having been scolded and beaten by parents, poor or dissatisfying test performance, conflict with friends, heavy course load and failure in selection processes.

**Table 1:** Relationship between parental educational level and social-economic, adverse family and college environments.

Exposure	Total (N = 5180) N(%)	Parental educational level				
		primary school or below	junior middle school	senior high school	university or above	P
Characteristic of participants						
Gender						<0.01
Males	2643(51.0)	248(62.3)	683(56.8)	1068(49.5)	644(45.2)	
Females	2537(49.0)	150(37.7)	519(43.2)	1088(50.5)	780(54.8)	
Age (M±SD)	21.32±2.20	22.29±3.34	21.45±2.15	21.38±2.00	20.84±2.00	<0.01
University type						<0.01
Key element	1557(30.1)	149(37.4)	390(32.4)	614(28.5)	404(28.4)	
Ordinary	3623(69.9)	249(62.6)	812(67.6)	1542(71.5)	1020(71.6)	
Only child						<0.01
Yes	3728(72.0)	90(22.6)	569(47.3)	1678(77.8)	1391(97.7)	
No	1452(28.0)	308(77.4)	633(52.7)	478(22.2)	33(2.3)	
Smoking						<0.05
Yes	482(9.3)	43(10.8)	115(9.6)	218(10.1)	106(7.4)	
No	4698(90.7)	355(89.2)	1087(90.4)	1938(89.9)	1318(92.6)	
Drinking						<0.01
Yes	1711(33.0)	152(38.2)	424(35.3)	703(32.6)	432(30.3)	
No	3469(67.0)	246(61.8)	778(64.7)	1453(67.4)	992(69.7)	
Disease affect						0.491
Yes	682(13.2)	56(14.1)	172(14.3)	272(12.6)	182(12.8)	
No	4498(86.8)	342(85.9)	1030(85.7)	1884(87.4)	1242(87.2)	
Insomnia						<0.01
Yes	1427(27.5)	132(33.2)	374(31.1)	577(26.8)	344(24.2)	
No	3753(72.5)	266(66.8)	828(68.9)	1579(73.2)	1080(75.8)	
Adverse family environment						
Poor family economic status						<0.01
Yes	1330(25.7)	216(54.3)	479(39.9)	504(23.4)	131(9.2)	
No	3850(74.3)	182(45.7)	723(60.1)	1652(76.6)	1293(90.8)	
Parental divorce						0.341
Yes	204(3.9)	9(2.3)	47(3.9)	90(4.2)	58(4.1)	
No	4976(96.1)	389(97.7)	1155(96.1)	2066(95.8)	1366(95.9)	
Poor parental relationship						0.197
Yes	229(4.4)	23(5.8)	60(5.0)	94(4.4)	52(3.7)	
No	4951(95.6)	375(94.2)	1142(95.0)	2062(95.6)	1372(96.3)	
Paternal or maternal unemployment						<0.01
Yes	533(10.3)	27(6.8)	119(9.9)	291(13.5)	96(6.7)	
No	4647(89.7)	371(93.2)	1083(90.1)	1865(86.5)	1328(93.3)	
Long time separate from family						<0.01
Yes	3338(64.4)	317(79.6)	866(72.0)	1391(64.5)	764(53.7)	
No	1842(35.6)	81(20.4)	336(28.0)	765(35.5)	660(46.3)	
Family internal conflicts						<0.01
Yes	1677(32.4)	162(40.7)	450(37.4)	676(31.4)	389(27.3)	
No	3503(67.6)	236(59.3)	752(62.6)	1480(68.6)	1035(72.7)	
Scolded and beaten by parents						<0.05
Yes	825(15.9)	66(16.6)	206(17.1)	304(14.1)	249(17.5)	
No	4355(84.1)	332(83.4)	996(82.9)	1852(85.9)	1175(82.5)	
Adverse college environment						
Major dissatisfaction						0.638
Yes	349(6.7)	22(5.5)	83(6.9)	153(7.1)	91(6.4)	
No	4831(93.3)	376(94.5)	1119(93.1)	2003(92.9)	1333(93.6)	
Test lose or dissatisfaction						<0.01
Yes	4153(80.2)	302(75.9)	995(82.8)	1706(79.1)	1150(80.8)	
No	1027(19.8)	96(24.1)	207(17.2)	450(20.9)	274(19.2)	
Conflict with friends						<0.05
Yes	3084(59.5)	227(57.0)	743(61.8)	1304(60.5)	810(56.9)	
No	2096(40.5)	171(43.0)	459(38.2)	852(39.5)	614(43.1)	
Heavy learning burden						<0.05
Yes	3261(63.0)	267(67.1)	787(65.5)	1336(62.0)	871(61.2)	
No	1919(37.0)	131(32.9)	415(34.5)	820(38.0)	553(38.8)	
Tension with teacher						0.162
Yes	679(13.1)	62(15.6)	172(14.3)	270(12.5)	175(12.3)	
No	4501(86.9)	336(84.4)	1030(85.7)	1886(87.5)	1249(87.7)	
Selection lose						<0.05
Yes	1279(24.7)	112(28.1)	327(27.2)	501(23.2)	339(23.8)	
No	3901(75.3)	286(71.9)	875(72.8)	1655(76.8)	1085(76.2)	
Transfer to another school or quit school						0.240
Yes	334(6.4)	38(7.5)	86(7.2)	122(5.7)	96(6.7)	
No	4846(93.6)	368(92.5)	1116(92.8)	2034(94.3)	1328(93.3)	
Entrance pressure						0.597
Yes	2124(41.0)	169(42.5)	498(41.4)	861(39.9)	596(41.9)	
No	3056(59.0)	229(57.5)	704(58.6)	1296(60.1)	828(58.1)	

Lower parental educational level was associated with depression in college students. Low family economic status, paternal or maternal unemployment, long periods spent apart from family, family conflicts, poor or dissatisfying test performance, conflict with friends, heavy course load and failure in selection processes were also associated with depression in college students after controlled gender, age, university type, only-child status, alcohol consumption, symptoms of ill health and insomnia (Table 2). These models explained 14.8% of the variation in predicted severity of depression. Additionally, a Sobel test was conducted for the significant mediated variables. Poor family economic status, paternal or maternal unemployment, long periods spent apart from family, family conflicts, poor or dissatisfying test performance, conflict with friends and heavy course load were all significant mediators of the relationship between parental educational level and depression in college students. Although failure in selection processes was a predictor of depression in college students, it was not a significant mediator.

**Table 2:** Association of college students’ depression with parental educational level, with and without adjustment for potential mediated adverse family and college environments.

Variables	Depressive score		
	b(SE)	P	P(Sobel)^a^
Parental educational level model(unadjusted)			
Parental educational level			
Primary school or below	2.007(0.342)	<0.01	
Junior middle school	0.992(0.236)	<0.01	
Senior high school	0.463(0.206)	<0.05	
Parental educational level (adjusted)^b^			
Parental educational level			
Primary school or below	0.948(0.360)	<0.01	
Junior middle school	0.094(0.247)	0.703	
Senior high school	0.079(0.199)	0.690	
Poor family economic status	0.761(0.196)	<0.01	<0.01
Paternal or maternal unemployment	0.649(0.266)	<0.05	<0.05
Long time separate from family	0.730(0.173)	<0.01	<0.01
Family internal conflicts	1.304(0.179)	<0.01	<0.01
Scolded and beaten by parents	0.376(0.228)	0.099	
Test lose or dissatisfaction	0.990(0.206)	<0.01	<0.05
Conflict with friends	0.683(0.169)	<0.01	<0.05
Heavy learning burden	1.136(0.171)	<0.01	<0.05
Selection lose	0.485(0.188)	<0.01	0.057

^a^ Sobel tests for mediation were significant (P < 0.05) in these models.

^b^ Model R^2^ for severity of depression score = 0.14

## Discussion

This study found that parental educational level is related to depression in college students. The relationship was more significant at lower levels of parental education. Low family economic status, paternal or maternal unemployment, long periods spent apart from family, family conflicts, poor or dissatisfying test performance, conflict with friends, heavy course load and failure in selection processes were related to depression in college students. All but failure in selection processes partially mediated the path between parental educational level and depression in college students.

Low parental educational levels are related to depressive symptoms exhibited by college students. A previous study suggested that parental education is correlated to depressive symptoms of children, as determined through a univariate analyses that was adjusted for age and puberty [15]. Our results also revealed the same finding. Bettina and his colleagues suggested that students from middle- or lower-class families reported more depressive symptoms [6]. This relationship may stem from the less access of college students from lower-class families to financial and psychological resources, such as parental affordance, liberal attitudes and good healthcare [8]. Our study emphasizes the importance of paying attention to college students whose parents achieved a lower level of education with the aim of preventing depression. It is also important to elucidate the relationships among factors that predict depression in college students from less-educated families. Moreover, this mediating effect supports our hypothesis that the parental educational level influences the risk of depression in college students due to their exposure to adverse family and college environments. In addition, a partially mediated effect was observed among the parental educational level, adverse family and college environments and college students’ depression. This finding is consistent with that reported by Evans, who found that SES-associated factors influence health by mediating the exposure to adverse environments [16].

Our study also has limitations. First, we collected samples from only one northeastern city. Although the colleges in Harbin enrolled students from all over China, the student population likely comprised mostly local students. Second, the parental educational level and adverse environments were evaluated using self-report questionnaires. The selected variable may have been subjective and unquantifiable and may exhibit both recall and response bias. In addition, because this survey was cross-sectional, we cannot determine the causal relationship among the parental educational level, adverse family and college environments and college students’ depression. Although we fully considered the potential influence of the family and college environments and controlled the potential confounders, unmeasured variables and confounders may exist. The data suggested that family and college environments are a potential mediator, but this finding was inconsistent and lacked statistical power[16]. Improving the environment appeared to not change the impact of the parental educational level on college students’ depressive symptoms.

## Conclusions

This study identified many family and college environment-related predictors of depression in college students form families with lower parental educational levels. Low family economic status, parental or maternal unemployment, long periods spent apart from family and family conflicts are all components of a negative family environment that mediate the relationship between parental educational achievements and depression in college students. Components of negative college environments include poor or dissatisfying test performance, conflict with friends and heavy course load. These factors have a mediating role between parental educational level and students’ depression.

To prevent depression in college students, it is important to support positive family and college environments and note the educational level of the students’ parents. Parents should pay attention to ensuring that college students are exposed to a good family environment. Colleges should establish psychological services to help students manage negative feelings after poor or dissatisfying test performance or conflicts with other students and decrease the feelings of stress associated with learning. Finally, future research should investigate different interventions for the prevention of depression in college students.

## Supporting Information

S1 Dataset Dataset of the subsamples. (RAR)

## References

[1] Goodman E, Slap GB, Huang B. The Public Health Impact of Socioeconomic Status on Adolescent Depression and Obesity. Adolescent Health. 2003; 93: 11. 10.2105/ajph.93.11.1844 PMC1448061 14600051

[2] Bayram N, Bilgel N. The prevalence and socio-demographic correlations of depression, anxiety and stress among a group of university students. Soc Psychiatry Psychiatr Epidemiol. 2008; 43: 8. 10.1007/s00127-008-0345-x 18398558

[3] Young CB, Fang DZ, Zisook S. Depression in Asian–American and Caucasian undergraduate students. Journal of Affective Disorders. 2010; 125: 1–3. 20303181 10.1016/j.jad.2010.02.124

[4] Zisook S, Trivedi MH, Warden D, Lebowitz B, Thase ME, Stewart JW, et al Clinical correlates of the worsening or emergence of suicidal ideation during SSRI treatment of depression: an examination of citalopram in the STAR*D study. Journal of Affective Disorders. 2009; 117: 1–2. 19217668 10.1016/j.jad.2009.01.002

[5] Xu Y, Chi X, Chen S, Qi J, Zhang P, Yang Y. Prevalence and correlates of depression among college nursing students in China. Nurse Education Today. 2014; 34, e7–e12. 10.1016/j.nedt.2013.10.017 24268639

[6] Piko BF, Fitzpatrick KM. Socioeconomic Status, Psychosocial Health and Health Behaviours among Hungarian Adolescents. European Journal of Public Health. 2007; 17:353–360. 17130141 10.1093/eurpub/ckl257

[7] Fors S, Lennartsson C, Lundberg O. Childhood living conditions, socioeconomic position in adulthood, and cognition in later life: exploring the associations. Journals of Gerontology. 2009; 64:750–757. 10.1093/geronb/gbp029 19420323

[8] Wirback T, Möller J, Larsson JO, Galanti MR, Engström K. Social factors in childhood and risk of depressive symptoms among adolescents–a longitudinal study in Stockholm, Sweden. International Journal for Equity in Health. 2014; 13:1–11. 25384415 10.1186/s12939-014-0096-0 PMC4243322

[9] Wu WC, Kao CH, Yen LL, Lee SH. Comparison of children's self-reports of depressive symptoms among different family interaction types in northern Taiwan. Bmc Public Health. 2007; 7:116 17584496 10.1186/1471-2458-7-116 PMC1919369

[10] Evans GW, Kantrowitz E. Socioeconomic status and health: The Potential Role of Environmental Risk Exposure. Annu. Rev. Public Health. 2002; 23. 10.1146/annurev.publhealth.23.112001.112349 11910065

[11] Eley TC, Liang H, Plomin R, Sham P, Sterne A, Willianmson K, et al Parental familial vulnerability, family environment, and their interactions as predictors of depressive symptoms in adolescents. Journal of the American Academy of Child and Adolescent Psychiatry. 2004; 43: 298–306. 15076263 10.1097/00004583-200403000-00011

[12] Liu XC Oda S, Peng X, Asai K. Life events and anxiety in chinese medical students. Social Psychiartry and psychiatric Epidemiology. 1997; 32: 63–67. 10.1007/BF00788922 9050346

[13] Beck AT, Beck RW. Screening depressed patients in family practice: A rapid technic. Postgraduate Medicine. 1972; 52: 81–85. 4635613 10.1080/00325481.1972.11713319

[14] Furukawa T A. Assessment of mood: Guides for clinicians. Journal of Psychosomatic Research. 2010; 68:581–589. 10.1016/j.jpsychores.2009.05.003 20488276

[15] Jackson B, Goodman E. Low Social Status Markers: Do They Predict Depressive Symptoms in Adolescence?. Race & Social Problems. 2011; 3:119–128. 22707986 10.1007/s12552-011-9047-1 PMC3373305

[16] Evans GW, Kantrowitz E. Socioeconomic status and health: The Potential Role of Environmental Risk Exposure. Annu. Rev. Public Health. 2002; 23. 10.1146/annurev.publhealth.23.112001.112349 11910065

